# DJ-1 protects against cell death following acute cardiac ischemia–reperfusion injury

**DOI:** 10.1038/cddis.2014.41

**Published:** 2014-02-27

**Authors:** R K Dongworth, U A Mukherjee, A R Hall, R Astin, S-B Ong, Z Yao, A Dyson, G Szabadkai, S M Davidson, D M Yellon, D J Hausenloy

**Affiliations:** 1 The Hatter Cardiovascular Institute, University College London, London, UK; 2 Department of Cell and Developmental Biology, Consortium for Mitochondrial Research, University College London, London, UK; 3 Department of Clinical Sciences, Faculty of Biosciences and Medical Engineering, Satellite Building V01, Universiti Teknologi Malaysia, Johor Bahru 81310 UTM, Malaysia; 4 Department of Medicine, University College London, London, UK

**Keywords:** DJ-1, PARK7, ischemia–reperfusion, cardioprotection, mitochondria

## Abstract

Novel therapeutic targets are required to protect the heart against cell death from acute ischemia–reperfusion injury (IRI). Mutations in the DJ-1 (PARK7) gene in dopaminergic neurons induce mitochondrial dysfunction and a genetic form of Parkinson's disease. Genetic ablation of DJ-1 renders the brain more susceptible to cell death following ischemia–reperfusion in a model of stroke. Although DJ-1 is present in the heart, its role there is currently unclear. We sought to investigate whether mitochondrial DJ-1 may protect the heart against cell death from acute IRI by preventing mitochondrial dysfunction. Overexpression of DJ-1 in HL-1 cardiac cells conferred the following beneficial effects: reduced cell death following simulated IRI (30.4±4.7% with DJ-1 *versus* 52.9±4.7% in control; *n*=5, *P*<0.05); delayed mitochondrial permeability transition pore (MPTP) opening (a critical mediator of cell death) (260±33 s with DJ-1 *versus* 121±12 s in control; *n*=6, *P*<0.05); and induction of mitochondrial elongation (81.3±2.5% with DJ-1 *versus* 62.0±2.8% in control; *n*=6 cells, *P*<0.05). These beneficial effects of DJ-1 were absent in cells expressing the non-functional DJ-1^L166P^ and DJ-1^Cys106A^ mutants. Adult mice devoid of DJ-1 (KO) were found to be more susceptible to cell death from *in vivo* IRI with larger myocardial infarct sizes (50.9±3.5% DJ-1 KO *versus* 41.1±2.5% in DJ-1 WT; *n*≥7, *P*<0.05) and resistant to cardioprotection by ischemic preconditioning. DJ-1 KO hearts showed increased mitochondrial fragmentation on electron microscopy, although there were no differences in calcium-induced MPTP opening, mitochondrial respiratory function or myocardial ATP levels. We demonstrate that loss of DJ-1 protects the heart from acute IRI cell death by preventing mitochondrial dysfunction. We propose that DJ-1 may represent a novel therapeutic target for cardioprotection.

Novel therapeutic strategies capable of protecting the heart against acute ischemia–reperfusion injury (IRI) are needed to reduce myocardial infarct (MI) size, preserve left ventricular (LV) systolic function and improve clinical outcomes in patients with ischemic heart disease (IHD), the leading cause of death and disability worldwide. Mitochondrial dysfunction induced by acute IRI is a major determinant of myocardial injury and cardiomyocyte cell death. Preservation of cardiomyocyte mitochondrial function in patients following IRI may therefore be expected to reduce cell death, prevent the onset of heart failure and ultimately improve long-term prognosis of IHD patients.

DJ-1 (also known as PARK7 (Parkinson disease (autosomal recessive, early onset) 7)) is a widely expressed and conserved mitochondrial protein that has been implicated in numerous pathologies, most notably in neurodegeneration where mutations in DJ-1 (e.g., the L166P mutation) ultimately result in an early-onset familial form of Parkinson's disease.^1^ Several experimental studies have elucidated important properties for DJ-1, including oxidative stress sensing, reactive oxygen species scavenging^2, 3, 4^ and preservation of normal mitochondrial function in the brain.^2, 5, 6^ Given its crucial role in maintaining mitochondrial function, DJ-1 represents a potential target for novel therapies for other pathologies involving mitochondrial dysfunction and cell death, including IRI. Indeed, animal studies have shown that DJ-1 ablation renders the brain more susceptible to cell death in the setting of stroke.^7^ Although DJ-1 is expressed in the heart,^1^ its role there is not well understood.

In the current study, we investigated the role of mitochondrial DJ-1 in the heart and its potential as a novel therapeutic target for protecting the heart against cell death following acute IRI.

## Results

### DJ-1 overexpression reduced HL-1 cell death from simulated IRI

**Figure 1:**
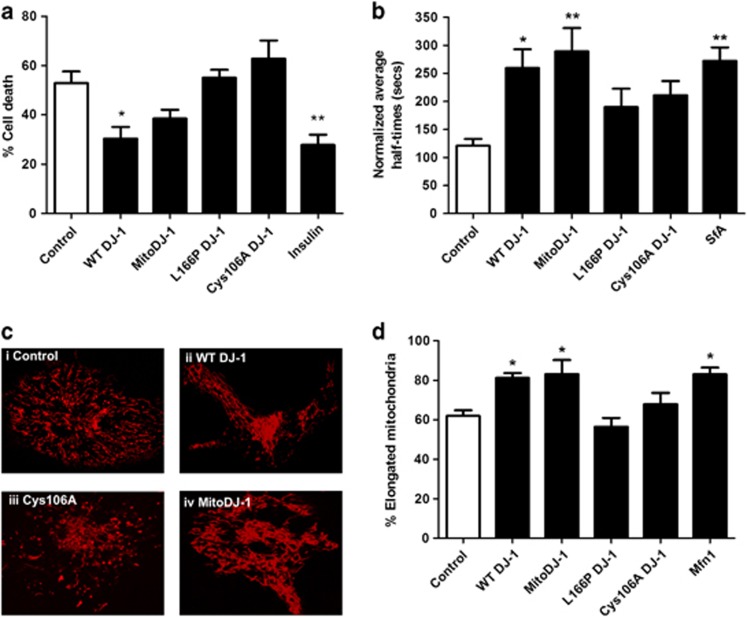
Assessment of the role of DJ-1 *in vitro*. Modulation of DJ-1 protein *in vitro* in HL-1 cells by transfection with WT and MitoDJ-1 and non-functional mutant DJ-1 (L166P and Cys106A). (**a**) Cell survival in response to simulated IRI, *n*=5/group. (**b**) MPTP opening in response to laser-induced oxidative stress, *n*≥5/group. (**c**) Representative images of TMRM-loaded mitochondria: (i) Control, (ii) WT-DJ (more elongated mitochondria), (iii) Cys106A and (iv) MitoDJ-1 (more elongated mitochondria). (**d**) Mitochondrial morphology, *n*≥5/group. Significance assessed by one-way ANOVA; significance indicated against comparison with control (white bars). *P<0.05, **P<0.01

Cell death was assessed in the HL-1 cardiac cell line in response to simulated IRI. WT DJ-1 overexpressing cells exhibited significantly lower cell death following simulated IRI (Figure 1a: cell death: WT DJ-1 30.4± 4.7% *versus* vector control 52.9±4.7%, *n*=5, *P<0.05) to a similar extent as the insulin-positive control. Expression of mitochondrial targeted DJ-1 (MitoDJ-1) also reduced cell death, although this was not statistically significant (Figure 1a: cell death: MitoDJ-1 38.5±3.5% *versus* vector control 52.9±4.7%, *n*=5, ^NS^*P*>0.05 (NS, not statistically significant)). Interestingly, expression of mutant DJ-1 did not affect cell death following simulated IRI (Figure 1a: cell death: L166P 55.1±3.2%, Cys106A 62.8±7.3% *versus* vector control 52.9±4.7% *n*=5, ^NS^*P*>0.05). Cell death in un-transfected time-matched normoxic control cells was <5.0%, and this was not significantly altered by transgene expression (data not shown).

### DJ-1 overexpression delayed mitochondrial permeability transition pore (MPTP) opening in HL-1 cells

MPTP opening is a critical mediator of cell death. DJ-1 overexpression significantly delayed MPTP opening in HL-1 cells (Figure 1b: normalized half-times: WT DJ-1 260±33 s *versus* vector control 121±12 s, *n*=6, *P<0.05). Expression of MitoDJ-1 also significantly delayed MPTP opening (Figure 1b: normalized half-times: MitoDJ-1 290±42 s *versus* vector control 121±12 s, *n*≥5, **P<0.01). Expression of DJ-1 mutants, L166P and Cys106A did not significantly affect MPTP opening times in this model (Figure 1b: normalized half-times: L166P 211±25 s, Cys106A 190±33 s *versus* vector control 121±12 s, *n*=6, ^NS^*P*>0.05).

### DJ-1 overexpression induced mitochondrial elongation in HL-1 cells

Mitochondrial morphology is an important indicator of cellular health and susceptibility to cell death; where mitochondrial elongation confers a cardioprotective phenotype. DJ-1 overexpression increased the proportion of cells displaying predominantly elongated mitochondria (Figure 1d: cells with elongated mitochondria: WT DJ-1 81.3±2.5% *versus* vector control 62.0± 2.8% *n*=6, *P<0.05). Expression of MitoDJ-1 also induced significant mitochondrial elongation (Figure 1d: cells with elongated mitochondria: MitoDJ-1 83.2±7.1% *versus* vector control 62.0± 2.8% *n*≥5, *P<0.05). Expression of DJ-1 mutants did not significantly affect mitochondrial morphology (Figure 1d: cells with elongated mitochondria: L166P 67.9±5.7%, Cys106A 56.4±4.5% *versus* vector control 62.0± 2.8% *n*=6, ^NS^*P*>0.05).

### Cardiac phenotype in DJ-1 WT and knockout (KO) mice

**Figure 2:**
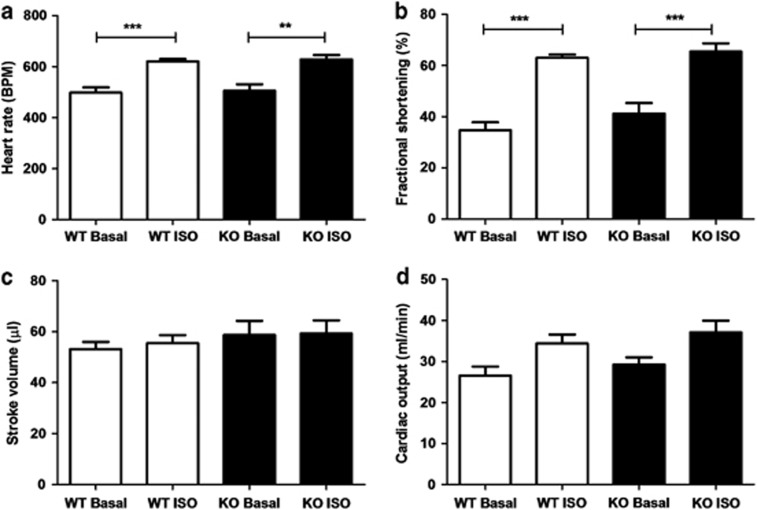
Echocardiographic phenotyping of DJ-1 WT and KO animals. (**a**–**d**) Isoproterenol (4 ng/g) treatment significantly increased the heart rate and fractional shortening in both DJ-1 WT and KO mice (*n*≥5/group; significance assessed by one-way ANOVA comparing WT Basal with WT ISO and KO Basal with KO ISO, where **P<0.01 and ***P<0.001). There were no significant differences in any cardiac parameter between DJ-1 WT and KO mice at baseline or in response to isoproterenol challenge (*n*≥5/group, significance assessed by one-way ANOVA comparing WT and KO, ^NS^*P*>0.05)

There were no significant differences in anterior and posterior LV wall dimensions during systole or diastole (examples of echocardiographic images are provided in Supplementary Figure S1). Isoproterenol stress yielded a significant increase in heart rate in both DJ-1 WT and KO animals (Figure 2a: heart rate in beats per minute: WT DJ-1 baseline 498.5±19.9 and isoproterenol 620.2±10.2, *n*=6, *P*<0.05; DJ-1 KO baseline 505±25.9 and isoproterenol 628.8±17.0, *n*=5, *P*<0.05) and fractional shortening (Figure 2b: percentage of fractional shortening: WT DJ-1 baseline 34.7±3.1 and isoproterenol 63.0±1.2, *n*=6, *P*<0.05; DJ-1 KO baseline 41.2±4.1 and isoproterenol 65.4±3.2, *n*=5, *P*<0.05). There were no differences in cardiac output or stroke volume between DJ-1 WT and KO mice at baseline or following isoproterenol challenge (Figures 2c and d).

### DJ-1 KO increased susceptibility to cell death from IRI *in vivo*

**Figure 3:**
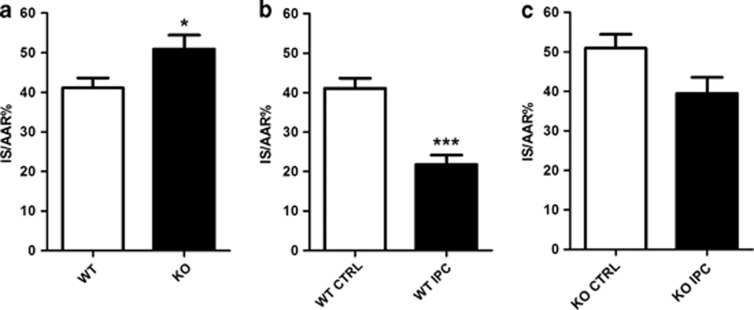
Susceptibility of DJ-1 KO to *in vivo* IRI. Infarct size following *in vivo* IRI in DJ-1 WT and KO mice. IS normalized to myocardial AAR to give IS/AAR%. (**a**) IS/AAR% in DJ-1 WT and KO mice subjected to standard IRI model of 45 min ischemia and 24 h reperfusion, *n*≥7/group. (**b** and **c**) IS/AAR% where standard IPC protocol of 5 min ischemia and 5 min reperfusion applied before main ischemic insult, *n*≥7/group. Significance assessed by unpaired *t*-test, *P<0.05, ***P<0.001. No statistical significance indicated as ^NS^*P*>0.05

DJ-1 KO mice exhibited a significant increase in cardiomyocyte cell death following *in vivo* IRI compared with DJ-1 WT ones (Figure 3a: infarct size %IS/AAR (infarct size/area-at-risk): DJ-1 KO 50.9±3.5% *versus* DJ-1 WT 41.1±2.5; *n*≥7/group, *P<0.05). Interestingly, DJ-1 KO mice appeared to be partially resistant to the MI size limiting effects of ischemic preconditioning (IPC) (Figures 3b and c: %IS/AAR in DJ-1 KO mice: control 50.9±3.5% *versus* IPC 39.4±4.1%, *n*=8/group, ^NS^*P*>0.05). The extent of cardioprotection afforded by IPC was ≈47% in WTs compared with only ≈23% in KOs. There were no significant differences in body weight, heart weight or area-at-risk between any treatment groups in these experiments.

### Calcium-induced MPTP opening in DJ-1 WT and DJ-1 KO mice

**Figure 4:**
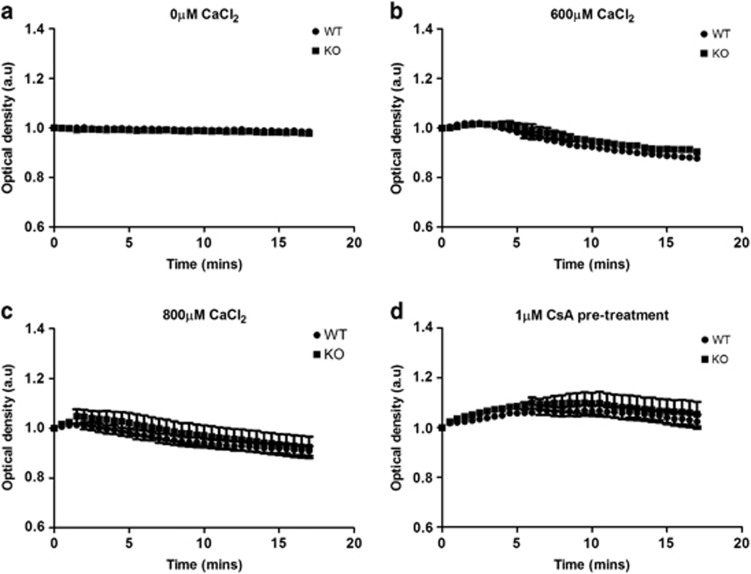
Calcium-induced mitochondrial swelling in isolated mitochondria. Mitochondria isolated from DJ-1 WT and KO hearts were subjected to calcium-induced mitochondrial swelling. Extent of mitochondrial swelling was assessed by optical density using spectrophotometer. (**a**) Baseline. (**b**) Calcium chloride (CaCl_2_) 600 *μ*M. (**c**) CaCl_2_ 800 *μ*M. (**d**) CsA pre-treatment at 1 *μ*M for 15 min before swelling assay using 600 *μ*M CaCl_2_. No apparent differences in mitochondrial swelling between DJ-1 WT and KO mitochondria, *n*=6/group

It was not possible to examine MPTP opening in primary isolated cardiomyocytes, as there was a strong trend to decreased mitochondrial membrane potential in DJ-1 KO hearts (Supplementary Figure S2), thereby precluding the use of the tetramethyl rhodamine methyl ester (TMRM)-based MPTP opening model. There were no differences in the swelling of mitochondria isolated from DJ-1 WT or DJ-1 KO hearts, suggesting that there was no difference in MPTP opening susceptibility (Figures 4a–c). As expected, ciclosporin A (CsA) treatment prevented calcium-induced mitochondrial swelling in both DJ-1 WT and DJ-1 KO mitochondria (Figure 4d).

### DJ-1 KO hearts display increased mitochondrial fragmentation

**Figure 5:**
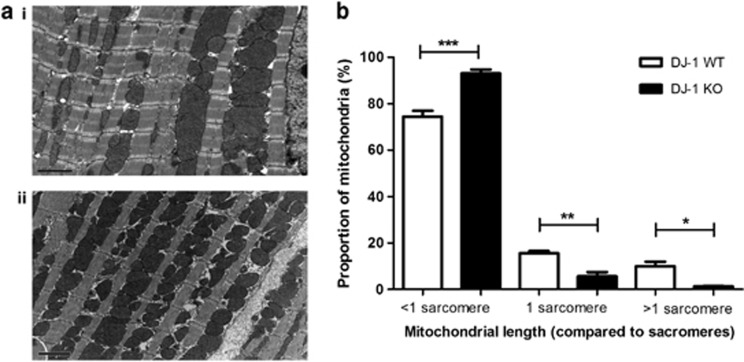
Mitochondrial morphology in DJ-1 WT and KO hearts at baseline. Mitochondrial morphology in DJ-1 WT and KO hearts was assessed using EM of hearts isolated at baseline. (**a**) Representative EM images showing mitochondrial morphology in DJ-1 WT (i) and KO (ii) hearts at baseline; scale bar=2 *μ*M. (**b**) Assessment of mitochondrial morphology by quantification of the proportion of mitochondria<1 sarcomere in length, equivalent to 1 sarcomere in length and >1 sarcomere in length, *n*≥3/group. Significance assessed by one-way ANOVA; significance indicated against comparison with DJ-1 WT control (white bars), *P<0.05, **P<0.01, ***P<0.001

At baseline, DJ-1 KO hearts exhibited a significantly greater proportion of short mitochondria (<1 sarcomere in length) and a concurrent decrease in longer mitochondria (≥1 sarcomere in length) (Figures 5a and b, *n*≥3/group). Sublethal ischemia resulted in increased proportions of short mitochondria in both DJ-1 WT and KO hearts. Interestingly, following ischemia, there was no significant difference in mitochondrial length between DJ-1 WT and DJ-1 KO hearts (see Supplementary Figure S3).

### Myocardial adenosine triphosphate (ATP) levels in DJ-1 WT and DJ-1 KO mice

**Figure 6:**
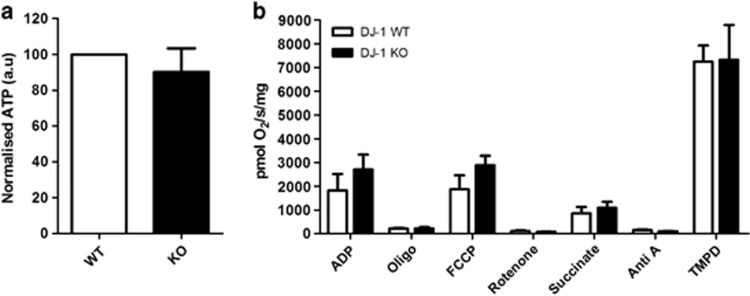
Functional characterization of DJ-1 WT and KO hearts. (**a**) Myocardial ATP assay in DJ-1 WT and KO hearts. ATP assessed using luminescence assay and normalized to DJ-1 WT baseline, *n*≥5/group. No significant difference in ATP levels between DJ-1 WT and KOs; significance assessed by unpaired *t*-test, ^NS^*P*>0.05. (**b**) Mitochondrial respiration assessed by oxygen flux readings for individual respiratory complexes in mitochondria isolated from DJ-1 WT and KO hearts, *n*≥5/group. No significant difference in mitochondrial respiratory function; significance assessed by one-way ANOVA, significance indicated against comparison with DJ-1 WT control (white bars), ^NS^*P*>0.05. Characterization of ATP levels and mitochondrial respiration in DJ-1 WT hearts is shown in Supplementary Figure S4

There were no significant differences in myocardial ATP levels between DJ-1 WT and DJ-1 KO mice at baseline (Figure 6a, *n*≥5/group, ^NS^*P*>0.05).

### Mitochondrial respiration in DJ-1 WT and DJ-1 KO mice

There were no differences in mitochondrial respiratory rates between the DJ-1 WT and DJ-1 KO hearts at baseline (Figure 6b, *n*≥5/group, ^NS^*P*>0.05).

The effect of DJ-1 genetic ablation on myocardial ATP levels and mitochondrial respiration could not be examined effectively in these animals, as a sublethal ischemic insult (of 20 min ischemia) did not induce a significant effect (see Supplementary Figure S4). The effect of an extended ischemic insult was not feasible given the clear effect of DJ-1 genetic ablation on cell survival indicated here.

## Discussion

In the present study, we demonstrate that mitochondrial DJ-1 may be an important therapeutic target for cardioprotection as evidenced by the following:(1) overexpression of WT DJ-1 but not its mutant forms reduced cell death following simulated IRI, delayed MPTP opening, and induced mitochondrial elongation in the HL-1 cardiac cell line; and (2) adult murine hearts deficient in DJ-1 were found to be more susceptible to cell death from IRI as evidenced by a larger MI size, a finding which was associated with increased mitochondrial fragmentation.

The beneficial effects of WT DJ-1 overexpression in HL-1 cells observed in our study appeared to be dependent on the ability of DJ-1 to dimerize and sense oxidation, as these effects were absent with the mutant forms of the protein, DJ-1^L166P^ (which is unable to dimerize) and DJ-1^Cys106A^ (in which Cys106 is resistant to oxidation). Dimerization and oxidation of DJ-1 are both known to be critical steps in its activation and mitochondrial translocation.^8, 9^ The beneficial effects of DJ-1 overexpression included reduced cell death from simulated IRI and protective effects on mitochondrial function, including delayed onset of MPTP opening (a critical mediator of cell death)^10^ and mitochondrial elongation (a change in mitochondrial morphology which has been reported to confer a cardioprotective phenotype).^11^ Although it should be noted that the cell death assay performed here could be improved by use of a more quantitative end point such as flow cytometry, the protective effect of DJ-1 overexpression against simulated IRI is also confirmed by the recent demonstration of a similar effect in H9c2 rat myoblasts.^12^

Adult mice deficient in DJ-1 were found to display no overt cardiac phenotype in terms of cardiac dimensions and function. This would suggest that DJ-1 does not appear to have a critical role in cardiac function under physiological conditions. The lack of any obvious cardiac phenotype such as LV hypertrophy therefore allowed us to examine the effect of DJ-1 deficiency on susceptibility to IRI, without any confounding myocardial pathology. Crucially, we found that hearts deficient in DJ-1 were more susceptible to cell death from acute IRI, as evidenced by a larger MI size. This finding suggests that endogenous DJ-1 may be an important target for cardioprotection. Interestingly, we also found that adult hearts lacking DJ-1 were resistant to the endogenous cardioprotective phenomenon of IPC, suggesting that DJ-1 may contribute to the signaling pathway underlying IPC. DJ-1 has also been reported to act as a mediator of hypoxic preconditioning in H9c2 rat myoblasts.^13^ In the brain, it has been reported that DJ-1 can increase the activity of the pro-survival kinase, Akt.^13, 14, 15^ Whether DJ-1 is required for the activation of Akt in the setting of IPC is an interesting possibility that remains to be explored.

Because DJ-1 overexpression in the HL-1 cell line delayed the onset of MPTP opening, we were expecting to observe an increased susceptibility to MPTP opening in cardiac mitochondria isolated from DJ-1 KO mice, but this was not the case. The reason for this discrepancy may relate to the experimental model used to induce and assess MPTP opening. In the HL-1 cell studies, MPTP opening was induced by oxidative stress in intact cardiomyocytes, whereas in the DJ-1 KO mice, MPTP opening was induced by calcium using isolated mitochondria. It was not possible to use the oxidative stress TMRM model of MPTP opening in cardiomyocytes isolated from DJ-1 KO hearts given the prominent trend to decreased mitochondrial membrane potential at baseline in DJ-1 KO cardiomyocytes compared with wild-type (WT) controls (Supplementary Figure S2). Many of the beneficial effects of DJ-1 in the brain relate to its anti-oxidant properties, and its ability to translocate to the mitochondria, and this may in part explain why we did not see the expected effects of DJ-1 ablation on MPTP opening in the isolated mitochondria model of calcium-induced MPTP opening. Indeed, it has recently been shown that DJ-1 protects the heart from oxidative stress resulting from transaortic banding.^16^

The overexpression of DJ-1 in HL-1 cardiac cells resulted in an increase in the proportion of cells displaying predominantly elongated mitochondria, a phenotype which we have previously reported to prevent IRI-induced mitochondrial fission and to be associated with cell survival.^11^ Interestingly, in the DJ-1 KO murine hearts we observed increased mitochondrial fragmentation. These findings suggest that mitochondrial DJ-1 can modify mitochondrial morphology, although the mechanism through which this is achieved is unclear. The protective effect of DJ-1 against mitochondrial fragmentation has also been reported in cultured SH-SY5Y cells.^17^ Despite the changes in mitochondrial morphology in DJ-1 KO hearts, we did not find any difference in mitochondrial respiration rates or myocardial ATP levels, suggesting that DJ-1 is not crucial for mitochondrial function at basal conditions. Whether we would have observed mitochondrial dysfunction under conditions of IRI remains to be determined. Interestingly, experimental studies of DJ-1 in the brain have also demonstrated that the DJ-1 ablation did not affect mitochondrial respiration in mouse embryonic fibroblast cells or primary isolated mitochondria from the cerebral cortex.^18^ It must be appreciated that DJ-1 may protect against cell death through other mechanisms, including (1) an anti-apoptotic pathway in which it sequesters death-domain-associated protein (Daxx) to disrupt Daxx-ASK1 (apoptosis signal-regulating kinase 1) regulated apoptosis^19^ or (2) mitophagy.^6^

In summary, we have found that in the adult murine heart DJ-1 may be a novel target for protecting the myocardium against cell death from acute IRI. Experimental studies have shown that the upregulation of DJ-1 by sodium 4-phenylbutyrate protected against acute IRI in the brain.^20, 21^ Whether this therapeutic approach is effective against acute myocardial IRI remains to be investigated.

## Materials and Methods

All animal experiments were conducted in accordance with the Animals (Scientific Procedures) Act 1986 published by the UK Home Office and the Guide for the Care and Use of Laboratory Animals published by the US National Institutes of Health 1996. All laboratory reagents were purchased from Sigma (Poole, UK) unless otherwise stated.

### HL-1 cell plasmid transfection

All *in vitro* experiments were conducted using the HL-1 cardiac cell line, cultured as described in published methods.^22^ HL-1 cells were transfected using Fugene6 (Roche, Basel, Switzerland) according to the manufacturer's instructions. Plasmids were: empty plasmid vector (RcCMV), mitochondrial red fluorescent protein (MtRFP), mitofusin 1 (Mfn1; Pcb6-MYC-Mfn1) (Professor L Scorrano, University of Padova, Padova, Italy), WT DJ-1 (WT DJ-1) (Dr. Z Yao and Dr. G Szabadkai, University College London, London, UK), WT DJ-1 with FLAG-tag sequence (pRK5 Flag DJ-1 WT) (Dr. Z Yao and Dr. G Szabadkai, University College London, London, UK), MitoDJ-1 (Dr. Z Yao and Dr. G Szabadkai, University College London, London, UK), L166P mutant DJ-1 (Dr. Z Yao and Dr. G Szabadkai, University College London, London, UK), Cys106A mutant DJ-1 (Professor P Kahle, University Clinics Tübingen, Tübingen, Germany), and plasmid-enhanced green fluorescent protein (Clontech, Mountain View, CA, USA). All cell experiments were conducted 24 h post transfection.

### Cell death following simulated IRI *in vitro*

HL-1 cells were subjected to simulated IRI comprising simulated ischemia for 12 h in ischemic buffer in an air-tight hypoxic chamber and simulated reperfusion for 1 h in normoxic buffer on removal from the hypoxic chamber. Cell death was assessed in 50 cells by propidium iodide staining, and PeGFP expression was observed using fluorescence microscopy (Nikon Eclipse TE200, Nikon, Kingston Upon Thames, UK). Control cell death was determined in un-transfected HL-1 cells incubated in oxygenated normoxic buffer for 13 h. Insulin (Invitrogen, Paisley, UK) treatment (275 U/ml) administered at the onset of reperfusion was used as a positive control.

### MPTP opening *in vitro*

MPTP opening in HL-1 cells was induced using laser-induced mitochondrial oxidative stress as previously published.^11, 23^ HL-1 cells were loaded with 3 μM TMRM for 15 min at 37 °C and imaged by time-scan confocal microscopy (Leica, Wetzlar, Germany). Time to MPTP opening was measured as the time taken to reach half maximum cytosolic TMRM fluorescence intensity of 15 cells for a minimum of five experiments. Sanglifehrin A (1 *μ*M), a known MPTP inhibitor, was used as a positive control.

### Mitochondrial morphology *in vitro* using confocal microscopy

HL-1 cell mitochondrial morphology was assessed using confocal microscopy (Leica) and MtRFP, as previously published.^11^ Ten randomly selected cells for a minimum of five independent experiments were imaged using a × 63 1/35 numerical aperture oil objective. Mitochondrial morphology was assessed by three blinded analyzers and defined as predominantly (>50%) elongated or fragmented. Mfn1 was used as a positive control.

### DJ-1 whole-body KO mice

Mice with whole-body genetic ablation of the DJ-1 gene (B6.Cg-*Park7*^*tm1Shn*^/J) were originally created by Goldberg *et al*,^24^ backcrossed onto C57BL/6 background for at least 12 generations. DJ-1 KO mice were always compared against true WT littermate controls (DJ-1 WT).

### Echocardiographic phenotyping

Cardiac phenotyping of DJ-1 WT and DJ-1 KO animals was performed by transthoracic two-dimensional echocardiography with Vivid 7 Dimension and 14 MHz probe (GE Healthcare, Hatfield, UK) as previously published^25^ to determine LV dimensions, heart rate, cardiac output, stroke volume and fractional shortening. Phenotyping was performed at baseline and 4 min following isoproterenol challenge (4 ng/g (Sigma) intraperitoneal bolus).

### Myocardial ischemia–reperfusion *in vivo*

DJ-1 WT and DJ-1 KO animals underwent *in vivo* myocardial IRI comprising open-chest surgery for occlusion of left anterior descending coronary artery followed by reperfusion. *Non-recovery*: Anesthesia was provided by ketamine 10 mg/ml (Vetalar, Boehringer Ingelheim, Bracknell, UK), xylazine 2 mg/ml (Rompun, Bayer, Newbury, UK) and atropine 0.06 mg/ml (Sigma) at 0.1 ml/10 g and 0.1 ml doses thereafter as required. *Recovery*: Anesthesia was by inhaled isoflurane (1.5–2.0% vaporized in 1.5 l/min oxygen). Analgesia was provided by buprenorphine (Vetergesic, Alstoe Animal Health, York, UK) 0.1 mg/kg intramuscular at 0, 6 and 24 h post surgery. Standard recovery IRI experiments comprised 15-min stabilization, 45-min ischemia and 24-h reperfusion. IPC protocol was 5-min stabilization, 5-min ischemia and 5-min reperfusion before the index ischemic event.

### MI size following *in vivo* IRI

At 24 h reperfusion, mice were anesthetized using non-recovery anesthetics as above, and hearts were rapidly extracted for staining with triphenyl-tetrazolium chloride and Evan's blue for determining MI size and AAR, respectively. Quantifications were performed on transverse heart slices using ImageJ planimetry (NIH Image, Bethesda, MD, USA), and MI size was expressed as percentage of AAR (%IS/AAR).

### Calcium-induced MPTP assay

Mitochondrial swelling was examined in mitochondria isolated from DJ-1 WT and DJ-1 KO hearts. Mitochondria were isolated by Trypsin (5 mg/ml) according to a modified protocol.^26^ Mitochondria were purified by differential centrifugation, and calcium-triggered mitochondrial-swelling assays were performed as described by Hafner *et al.*^27^ Briefly, calcium chloride (600 or 800 *μ*M) was added to mitochondria, and optical density was measured by spectrophotometry (FLUOstar Omega, BMG Labtech, Cary, NC, USA) for 17 min. Treatment with the known MPTP inhibitor, CsA (1.0 *μ*M), was used as a positive control.

### Mitochondrial morphology by electron microscopy (EM)

DJ-1 WT and DJ-1 KO hearts at baseline or after 20-min ischemia (*n*≥3/group) were prepared for EM as previously published and imaged using a Jeol 1010 electron microscope (Jeol Ltd, Welwyn Garden City, UK).^11^ Ten images were taken for each heart. Mitochondrial elongation was assessed by comparison of interfibrillar mitochondria (IFM) to sarcomere length. Analysis was performed by two blinded analyzers on a total of 500–600 IFM.

### Myocardial ATP assay

The left ventricles from DJ-1 WT and DJ-1 KO hearts were homogenized in PBS containing protease and phosphatase inhibitors and EDTA (pH 8.0) (Thermo Scientific, Epsom, UK) 1 ml/0.1 g of tissue. Myocardial ATP levels were measured using ATP bioluminescence assay kit (Sigma) in accordance with the manufacturer's instructions where luminescence was read every 5 s for 2 min (FLUOstar Omega, BMG Labtech).

### Mitochondrial respiration assay

Mitochondria were isolated from DJ-1 WT and DJ-1 KO hearts as described above. Oxygen consumption rates were measured using a high-resolution respirometer equipped with a Clark oxygen sensor (Oroboros Oxygraph, Paar KG, Graz, Austria).

### Statistics

All values are expressed as mean±S.E.M. Data were analyzed by one-way analysis of variance (ANOVA) and Tukey's multiple-comparison *post-hoc* test or unpaired *t*-test where indicated. Differences were considered significant when *P<0.05.

## Abbreviations

ATP: adenosine triphosphate
CsA: ciclosporin A
Daxx: death-domain-associated protein
EM: electron microscopy
IHD: ischemic heart disease
IPC: ischemic preconditioning
IRI: ischemia–reperfusion injury
IS/AAR: infarct size/area-at-risk
KO: knockout
LV: left ventricular
Mfn1: mitofusin 1
MitoDJ-1: mitochondrial targeted DJ-1
MI: myocardial infarct
MPTP: mitochondrial permeability transition pore
MtRFP: mitochondrial red fluorescent protein
NS: not statistically significant
TMRM: tetramethyl rhodamine methyl ester
WT: wild type
