# Supplementary material for: DJ-1 protects against cell death following acute cardiac ischemia–reperfusion injury
Source: Cell Death Dis. 2014 Feb 27;5(2):e1082–. doi: 10.1038/cddis.2014.41 (PMC3944257; doi:10.1038/cddis.2014.41)
Supplement: Supplementary Information [file cddis201441x1.doc]

**Mitochondrial DJ-1 protects the heart against acute ischemia-reperfusion injury**

Dongworth et al.

**Supplemental Material**

**Methods**

All animal experiments were conducted in accordance with the Animals (Scientific Procedures) Act 1986 published by the UK Home Office and the Guide for the Care and Use of Laboratory Animals published by the US National Institutes of Health 1996. All laboratory reagents were purchased from Sigma, UK unless otherwise stated.

**HL-1 cell plasmid transfection:** All *in vitro* experiments were conducted using the HL-1 cardiac cell line. HL-1 cells were cultured as described in the literature ^1^, under sterile conditions in Claycomb media (SAFC Biosciences, UK) supplemented with norepinephrine 100μM, fetal bovine serum (FBS) 10% (SAFC Biosciences, UK), L-glutamine 4mM (Sigma, UK), Penicillin 500IU/ml and Streptomycin 5µg/ml (Invitrogen, UK). Cells were incubated at 37ºC, 95% O_2_/5% CO_2_, 90% humidity on fibronectin-coated culture flasks for maintenance and fibronectin-coated glass coverslips (VWR International, UK) for all experiments.

**Cell death following simulated IRI *in vitro:*** Assay as described previously ^2^, where simulated ischemia was for 12 hours by incubation of HL-1 cells in ischemic buffer comprising (in mmol/L) KH_2_PO_4_ (1.0), NaHCO_3_ (10.0), MgCl_2_.6H_2_O (1.2), NaHEPES (25.0), NaCl (74.0), KCl (16.0), CaCl_2_ (1.2) and sodium lactate (20.0) at pH 6.2, bubbled with 95% N_2_/ 5% CO_2_. Cells were placed in an airtight modular incubation chamber (Billups-Rothenberg, USA) gassed with 95% N_2_/ 5% CO_2_ to evacuate O_2_ and stored in cell incubator as above. Simulated reperfusion was for 1 hour by incubation of cells in oxygenated normoxic buffer comprising (in mmol/L) KH_2_PO4 (1.0), NaHCO_3_ (10.0), MgCl_2_.6H_2_O (1.2), NaHEPES (25.0), NaCl (98.0), KCl (3.0), CaCl_2_ (1.2), d-glucose (10.0), Na pyruvate (2.0) at pH 7.4, bubbled with 5% CO_2_/ 95% O_2_.

PeGFP expression was used to confirm successful cell transfection. Propidium iodide 2μl (Invitrogen, UK) incorporation was used to visualize cell necrosis. Cell death was determined as number of dead transfected cells (propidium iodide and PeGFP positive) as a percentage of total transfected cells (PeGFP positive). Fifty cells were counted in a randomly selected field of view using a fluorescence microscopy (Nikon Eclipse TE200, Nikon, Europe). Control cell death was assessed in un-transfected HL-1 cells incubated in oxygenated normoxic buffer for 12 + 1 hours. Insulin (275U/ml) (Invitrogen, UK) applied at the onset of reperfusion treatment was used as a positive control for reduced cell death. This cell death assay was undertaken in a randomized manner and analysis of cell death was quantified by two analyzers blinded to the treatment groups.

**Mitochondrial permeability transition pore opening *in vitro:*** MPTP opening was assessed in HL-1 cells 24 hours post-transfection using an oxidative stress model as published previously ^2^. HL-1 cells were loaded with tetramethyl rhodaminemethyl ester (TMRM) 3μM for 15 minutes at 37ºC. For imaging, cells were incubated in Krebs-HEPES buffer comprising (in mmol/L) NaCl (118.0), NaHCO_3_ (25.0), d-Glucose (11.0), KCl (4.7), MgSO_4_.7H_2_O (1.2), KH_2_PO_4_ (1.2), CaCl_2_.2H_2_O (1.8) and HEPES (10.0). Cells were imaged by time-scan confocal microscopy (Zeiss, Germany) with 40X oil immersion, quartz objective lens (Plan-Neofluar NA 5 1.3, Nikon, Europe), simultaneous 488nm and 543nm excitation with emissions recorded at 505–53 nm and >560nm. Time to MPTP opening was measured as the time taken to reach half maximum cytosolic TMRM fluorescence intensity. Fifteen PeGFP expressing cells were randomly selected for MPTP opening, repeated for a minimum of five experiments (n≥5). Treatment with the MPTP inhibitor Sanglifehrin A (SfA) 1 μM for 10 minutes was used as a positive control to confirm mitochondrial membrane potential changes reflected MPTP opening.

**Mitochondrial morphology *in vitro* using confocal microscopy:** Mitochondrial morphology was assessed in HL-1 cells 24 hours post-transfection using MtRFP expression, as published previously ^2^. For imaging cells were incubated in Krebs-HEPES buffer as detailed above. Cells were imaged with a Zeiss 50 CLSM confocal microscope (Zeiss, Germany) using 63X oil immersion quartz objective lens (Plan Apochromat NA 1.3, Nikon, Europe) and HeNe laser (543nm emission and 560nm long pass filter). Ten cells per treatment group were randomly imaged for a minimum of 5 experiments (n≥5). Mitochondrial morphology was assessed by three blinded operators and defined as predominantly elongated or fragmented. Mfn1 expression was used as a positive control for mitochondrial elongation.

**DJ-1 whole-body knockout mice:** Mice with whole-body genetic ablation of the DJ-1 gene were as previously described in the literature ^3^ and were kindly donated by Dr F Giorgini and Dr R Mariaelena (University of Leicester, UK). Mice were bred in-house as heterozygous crosses to produce DJ-1 WT (+/+), HET (+/-) and KO (-/-) mice such that all experiments were conducted on true WT littermate controls. All animals were genotyped for the DJ-1 gene and experiments conducted on DJ-1 WT and KO animals. *Genotyping:* Genomic DNA samples from ear samples were processed using Qiagen DNeasy kit (Qiagen, UK) according to manufacturer’s instructions. PCR was conducted using the Qiagen Taq polymerase PCR kit (Qiagen, UK) with addition of the following primers: WT-forward: 5' GATCCAGTGCTCTTAGCCACAGACTA 3’; WT-reverse: 5’GCATGACATCCACAGGAATCACT 3’; KO reverse: 5’GGATCAATTCTCTAGAGCTCGCTGATCA 3’ (Eurofins MWG Operon, Germany). Analysis of PCR products was by gel electrophoresis using a 1.5% agarose gel containing SYTO60 red fluorescence nucleic acid stain (Molecular Probes Invitrogen, UK) for visualizing DNA products using a fluorescence scanner (Odyssey Imaging System, LI-COR Biosciences, UK). Expected sizes of PCR products were: WT 250bp, KO 500bp and HET 250bp and 500bp.

**Echocardiographic phenotyping**: Cardiac phenotyping was performed as described previously ^4^ using transthoracic echocardiography echocardiography (Vivid 7 Dimension™, GE Healthcare, Bedford, UK) using a 14 MHz probe recording at a depth of 0-1 cm. Mice were anesthetized with 1.5% isoflurane and placed in the supine position for imaging. Left ventricular (LV) end diastolic and systolic dimensions were determined from a parasternal short-axis view at the papillary muscle level using time-motion (M) mode. Measurements were taken of the internal dimension of the cavity using the leading-edge-to-leading-edge convention. The thickness of the LV anterior and posterior walls was also determined. Aortic blood flow velocities were determined by pulsed-wave Doppler the in aortic arch before the bifurcation of the right carotid artery. The direction of blood flow was confirmed by colour Doppler. Stroke volume was calculated as the product of the velocity time integral and the vessel cross-sectional area (π x [0.5 x diameter]^2^). Pilot studies in mice of this age showed the aortic diameter to be 1.34 mm, thus a cross sectional area of (0.67)^2^ x π was assumed for all animals. The peak aortic blood flow velocity was measured as the average maximum velocity from six velocity-time traces. Heart rate was determined by measuring the time between six consecutive cycles from the start of each Doppler trace. Cardiac output was calculated as the product of heart rate and stroke volume. Percentage fractional shortening was calculated as the (LV end diastolic diameter - LV end systolic diameter) divided by LV end diastole diameter multiplied by 100.

**Myocardial ischaemia-reperfusion *in vivo:*** Mice were anaesthetized (as detailed below) and surgical depth of anesthesia continually confirmed by loss of pedal reflex. Core body temperature was maintained at 36.5 ± 0.5°C. Cardiac monitoring by ECG was carried out using a three-lead setup attached subcutaneously to the limbs (Powerlab linked to Chart 7 software, AD Instruments, UK). Ventilation was provided by connection to a small animal ventilator (MiniVent 845, Hugo Sachs Electronik, Germany) supplied with oxygen. A thoracotomy was performed to provide access to the left ventricle and the left anterior descending (LAD) coronary artery was occluded and subsequently reperfused using 8-0 prolene suture (Ethicon, UK) and custom made snare (polyethylene-50 tubing, VWR International, UK). Ischemic and reperfusion durations for each experiment were as detailed below. LAD suture was left in place to permit subsequent identification of the area-at-risk (AAR).

**Cell death following *in vivo* IRI:** Mice were anaesthetized with non-recovery anesthetics and hearts rapidly extracted. The aorta was rapidly cannulated to allow retrograde perfusion of the coronary system. Hearts were washed by perfusion of 1ml saline. Infarct assessment was by perfusion of triphenyl-tetrazolium chloride (TTC), approximately 5ml pre-warmed to 37ºC. The LAD suture was then re-occluded and Evans blue dye perfused to stain the non-AAR. Hearts were stored at -20ºC for 24 hours prior to slicing into 5 transverse sections using a sharp scalpel. Heart slices were briefly washed in distilled washed before being incubated in paraformaldehyde for 2 hours. Slices were prepared for scanning by dissection of right ventricle and placed in a custom-made acrylic block in a pool of paraformaldehyde. Images were taken at 1200dpi using a Epson scanner (Epson, UK). Analysis was performed by planimetry using ImageJ software (NIH Image, USA) to quantify infarct size (IS/LV%) and AAR (AAR?LV%) to allow final expression of infarct size controlled to AAR (IS/AAR%).

**Mitochondrial membrane potential:** Mitochondrial membrane potential was assessed in isolated cardiomyocytes. Hearts were first prepared by Langendorff perfusion of oxygenated perfusion buffer (PB1) consisting of (in mM) NaCl (113), KCl (4.7), KH_2_PO_4_ (0.6), MgSO_4_.7H_2_O (1.2), Na_2_HPO_4_ (0.6), NaHCO_3_ (12), KHCO_3_ (10), phenol red (0.032), HEPES (4.2), taurine (30), glucose (5.5), 2,3–butanedione monoxime (10), pH 7.4, 37ºC at 5ml/min for 5 minutes followed by perfusion of digestion buffer (PB1 supplemented with 12.5μM CaCl_2_; 5x10^-3^U/ml DNase1 (New England Biolabs, USA); 0.14mg/ml Trypsin 2.5% (Invitrogen, UK) and 0.5mg/ml collagenase, 0.2mg/ml Liberase TM (Roche,UK) for 13 minutes (DJ-1 KO) or 22 minutes (DJ-1 WT). Digestion was stopped by addition of stopping buffer 1 (SB1: PB1 with 10% FBS (Invitrogen, UK), CaCl_2_ 12.5 μM) for 10 minutes and stopping buffer 2 (SB2: PB1, 5% FBS, CaCl_2_ 12.5μM) for 10 minutes. Calcium was then reintroduced by resuspension of the cell pellet in SB2 buffer containing 62.5μM, 112μM, 212μM, 500μM, 1mM CaCl_2_ serially for 10 minutes. Cell were then re-suspended in plating media (standard M-199 medium supplemented with 2mg/ml BSA, 0.66mg/ml creatine, 0.662mg/ml taurine, 0.322mg/ml carnitine, 50U/ml penicillin, 25μM Blebbistatin (Enzo Life Sciences, UK)) and added to laminin-coated coverslips pre-incubated at 37ºC, 95% O_2_/ 5% CO_2_.

For imaging, isolated cardiomyocytes were incubated with TMRM 50nM in Hanks balanced salt solution (HBSS) (in mM) [NaCl (156), KCl (3), MgSO_4_.7H_2_O (2), K_2_HPO_4_ (1.25), CaCl_2_ (2), HEPES (10), D-glucose (10mM) to 100ml dH_2_O pH 7.35] as described in the literature ^5, 6^. Each coverslip of cardiomyocytes was imaged 10 times with Zeiss 50 CLSM confocal microscope (Zeiss, Germany) with HeNe laser at 3%.

**Calcium-induced mitochondrial swelling assay:** Mitochondrial swelling was examined in mitochondria isolated from hearts prepared on Langendorff as above. Mitochondrial isolation was modified from a previously published method ^7^. Ventricles were then dissected and homogenized using a Potter-Elveheim glass homogeniser in CP1 buffer containing (in mM) KLC (100), Mops (50), MgSO_4_ (5.0), EGTA (1.0), ATP (1.0), pH 7.4 at a concentration of 1ml/0.1g of tissue. Mitochondria were isolated by incubation of ventricular homogenate in 1ml/g Trypsin (5mg/ml) for 10 minutes on ice. The trypsin was quenched by addition of CP2 buffer (CP1 plus 2mg/ml fatty acid free BSA), and homogenate spun at 500 x g for 10 minutes at 4°C. The supernatant was then re-spun at 1000 x g for 10 minutes at 4°C, before the mitochondrial pellet was recovered following a centrifugation at 5000 x g for 10 minutes at 4°C. The mitochondria were washed once in CP2 buffer before finally being re-suspended in mitochondrial swelling buffer containing (in mM) sucrose (200), HEPES (10), KH_2_PO_4_ (5), EGTA (10), succinate (10), Rotenone (1.5), pH 7.3.

Calcium-triggered mitochondrial swelling assays were performed as described by Hafner et al.^8^. Briefly, mitochondrial swelling was triggered by the addition of either 600 μM or 800 μM of CaCl_2_ to 0.5mg/ml isolated mitochondria. Mitochondrial swelling was monitored by optical density measured by FLUOstar Omega spectrophotometer (BMG Labtech, USA) at 540nm for 17 minutes. CsA treatment was 1 µM for 15 minutes prior to swelling assay. Mitochondria were pooled from 6 hearts for both WT and KO.

**Mitochondrial morphology *in vivo* by electron microscopy:** Hearts were collected following stabilization (25 minutes) or ischemia (5 minutes stabilization, 20 minutes ischemia). Fixed hearts were prepared for EM by isolation of a 2mm transverse slice, 3mm from the apex of the heart, as described previously ^2^. Slices were fixed in osmium (OsO_4_ 1% in PBS, 0.1M pH 7.3,) at 4◦C for 90 minutes prior to washing in PBS (0.1M pH 7.4) and Enbloc staining with uranyl acetate (0.5% in dH_2_O) for 30 minutes at 4◦C. Following washing in dH_2_O, heart slices were dehydrated in a graded ethanol-water series and infiltrated with Agar-100 resin overnight (Agar Scientific, UK). Semi-thin sections were cut at 1µm and mounted on glass slides and stained with toluidine blue 1% in dH_2_O for light microscopy. Ultrathin sections were cut at 70-80nm using a diamond knife on a Reichert Ultra-cut E microtome (Reichert Microscope Services, USA). Sections were collected on 200 mesh copper grids, stained with uranyl acetate and lead citrate. Ultra-thin sections were viewed with Jeol 1010 transition electron microscope (Jeol Ltd, UK).

**Myocardial ATP assay:** Heart samples were prepared on the Langendorff apparatus after which left ventricles were dissected and homogenized in ice-cold conditions in PBS buffer containing protease and phosphatase inhibitor cocktails and EDTA (pH 8.0) (Thermo Scientific, Epsom, UK) 1ml/0.1g of tissue. Myocardial ATP levels were measured using an ATP bioluminescence assay kit (Sigma, UK) in accordance with manufacturer’s instructions. 96 well plates were prepared with ATP assay mix diluted 1:25 in supplied buffer. Heart homogenates were added to ATP assay mix and luminescence read every 5 seconds for 2 minutes using FLUOstar Omega microplate reader (BMG Labtech, USA).

**Mitochondrial respiration assay:** Mitochondria were isolated as described above. Rates of oxygen consumption by intact mitochondria were measured using a high-resolution respirometer (Oroboros Oxygraph, Paar KG, Austria) equipped with a Clark oxygen sensor and analyzed with DATGRAPH Analysis software (OROBOROS, Austria). A Hamilton microsyringe was used to add substrates/inhibitors. Substrates used were malate 10mM, pyruvate 10mM, glutamate 10mM. 0.5mM ADP was added to measure State 3 respiration. After a steady State 4 respiration was achieved, oligomycin was added to a final concentration of 2.5µM to assess leak respiration. FCCP was added to a final concentration 2uM to assess uncoupling. Rotenone was added to final concentration 0.5uM to measure complex I inhibition, followed by Succinate 100µM to assess complex II respiration. Antimycin A 5uM was added to inhibit complex III and then ascorbate (20 µM) and TMPD (5 µM) were added to support complex IV respiration.

**Results**

**
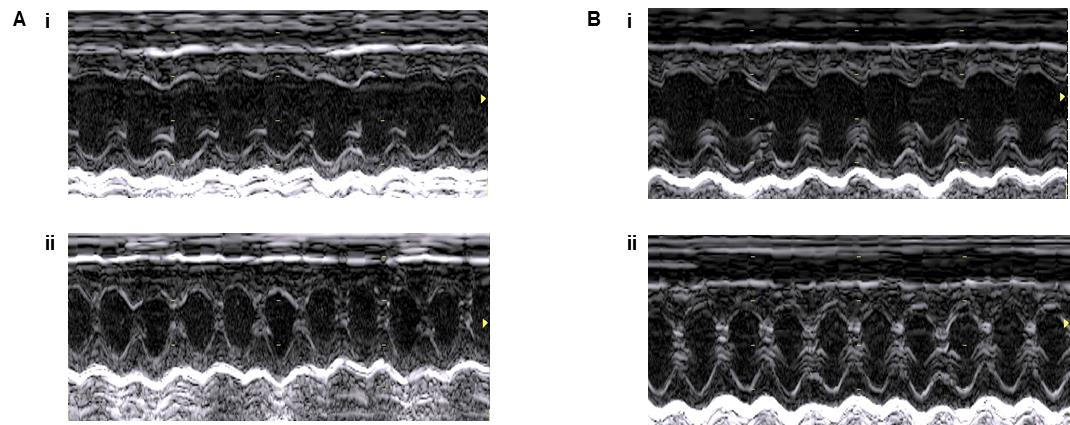
**

**S1**: **Echocardiographic phenotyping of DJ-1 WT and KO animals**

Echocardiography of DJ-1 WT and KO mice at baseline and following isoproterenol (4ng/g).

A-B. Representative echocardiography images of WT (A) and KO (B) at baseline (i) and following isoproterenol (ii).

**S2**: **Mitochondrial membrane potential in DJ-1 WT and KO cardiomyocytes**

Assessment of mitochondrial membrane potential by TMRM fluorescence at baseline in cardiomyocytes isolated from DJ-1 WT and KO hearts, n=3/group; statistical significance assessed by unpaired t-test, ^NS^P>0.05.


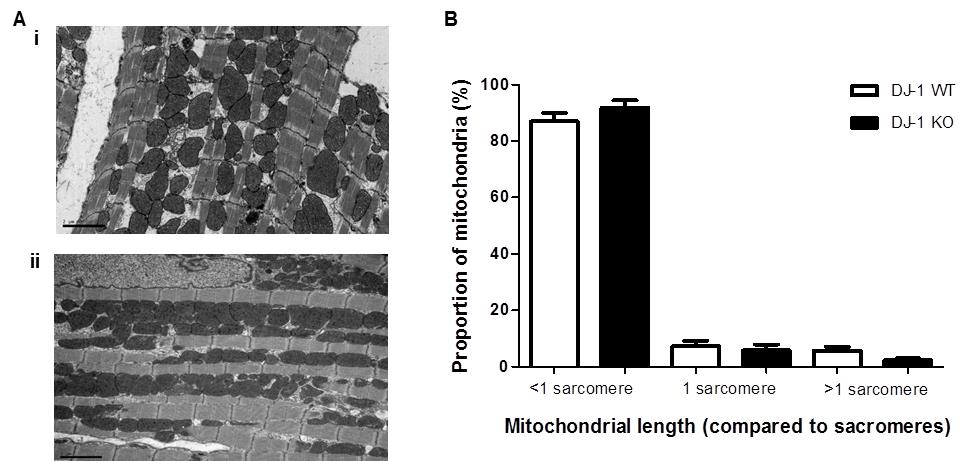


**S3: Mitochondrial morphology in DJ-1 WT and KO hearts following ischemia**

Mitochondrial morphology in DJ-1 WT and KO hearts was assessed using electron microscopy of hearts isolated following sublethal ischemia (20 minutes) (20 minutes ischemia).

A. Representative EM images showing mitochondrial morphology in DJ-1 WT (i) and KO (ii) hearts following ischemia; scale bar 2 µm. B. Assessment of mitochondrial morphology by quantification of the proportion of mitochondria <1 sarcomere in length, equivalent to 1 sarcomere in length and >1 sarcomere in length, n≥3/group. ^NS^P<0.05.

**S4: Functional characterization of DJ-1 WT hearts upon sublethal ischaemia**

Characterization of ATP levels and mitochondrial respiration in DJ-1 WT hearts at baseline and following a sublethal (20 minute) ischaemic insult. A. Myocardial ATP assay in DJ-1 WT hearts. ATP assessed using luminescence assay and normalized to DJ-1 WT baseline, n≥5/group. No significant difference in ATP levels between DJ-1 WT hearts at baseline and following ischaemia; significance assessed by unpaired t-test, ^NS^P>0.05. B. Mitochondrial respiration assessed by oxygen flux readings for individual respiratory complexes in mitochondria isolated from DJ-1 WT hearts at baseline and following ischaemia, n≥5/group. No significant difference in mitochondrial respiratory function; significance assessed by one-way ANOVA, significance indicated against comparison with DJ-1 WT basal (white bars), ^NS^P>0.05.

**References:**

(1) Claycomb WC, Lanson NA, Jr., Stallworth BS, Egeland DB, Delcarpio JB, Bahinski A, Izzo NJ, Jr. HL-1 cells: a cardiac muscle cell line that contracts and retains phenotypic characteristics of the adult cardiomyocyte. *Proc Natl Acad Sci U S A.* 1998;95(6):2979-84.

(2) Ong SB, Subrayan S, Lim SY, Yellon DM, Davidson SM, Hausenloy DJ. Inhibiting mitochondrial fission protects the heart against ischemia/reperfusion injury. *Circulation.* 2010;121(18):2012-22.

(3) Goldberg MS, Pisani A, Haburcak M, Vortherms TA, Kitada T, Costa C, Tong Y, Martella G, Tscherter A, Martins A, Bernardi G, Roth BL, Pothos EN, Calabresi P, Shen J. Nigrostriatal dopaminergic deficits and hypokinesia caused by inactivation of the familial Parkinsonism-linked gene DJ-1. *Neuron.* 2005;45(4):489-96.

(4) Lim SY, Hausenloy DJ, Arjun S, Price AN, Davidson SM, Lythgoe MF, Yellon DM. Mitochondrial cyclophilin-D as a potential therapeutic target for post-myocardial infarction heart failure. *J Cell Mol Med.* 2011;15(11):2443-51.

(5) Duchen MR, Surin A, Jacobson J. Imaging mitochondrial function in intact cells. *Methods Enzymol.* 2003;361:353-89.

(6) Davidson SM, Yellon D, Duchen MR. Assessing mitochondrial potential, calcium, and redox state in isolated mammalian cells using confocal microscopy. *Methods Mol Biol.* 2007;372:421-30.

(7) Rosca MG, Vazquez EJ, Kerner J, Parland W, Chandler MP, Stanley W, Sabbah HN, Hoppel CL. Cardiac mitochondria in heart failure: decrease in respirasomes and oxidative phosphorylation. *Cardiovasc Res.* 2008;80(1):30-9.

(8) Hafner AV, Dai J, Gomes AP, Xiao CY, Palmeira CM, Rosenzweig A, Sinclair DA. Regulation of the mPTP by SIRT3-mediated deacetylation of CypD at lysine 166 suppresses age-related cardiac hypertrophy. *Aging (Albany NY).* 2010;2(12):914-23.
